# Relationship between nosocomial infections and coronavirus disease 2019 in the neurosurgery unit: clinical characteristics and outcomes from a Chinese Tertiary-Care Hospital

**DOI:** 10.1186/s12879-022-07845-x

**Published:** 2022-11-11

**Authors:** Shuang-Jun Pan, Yong Hou, Yu-Pei Yang, Geng-Ge Wang, Xiao-Yan Chen, Wei-Yang Qian, Tao-Hsin Tung, Xiao-Ming Hu

**Affiliations:** 1 Department of Neurosurgery, Taizhou Hospital of Zhejiang Province Affiliated to Wenzhou Medical University, Linhai, 317000 Zhejiang China; 2 Department of Hematology, Taizhou Hospital of Zhejiang Province Affiliated to Wenzhou Medical University, Linhai, 317000 Zhejiang China; 3 Hospital Infection Control Department, Taizhou Hospital of Zhejiang Province Affiliated to Wenzhou Medical University, Linhai, 317000 Zhejiang China; 4 Evidence-Based Medicine Center, Taizhou Hospital of Zhejiang Province Affiliated to Wenzhou Medical University, Linhai, 317000 Zhejiang China; 5 Key Laboratory of Minimally Invasive Techniques & Rapid Rehabilitation of Digestive System Tumor of Zhejiang Province, Taizhou Hospital of Zhejiang Province Affiliated to Wenzhou Medical University, Linhai, 317000 Zhejiang China

**Keywords:** Nosocomial infections, COVID-19, Neurosurgery

## Abstract

**Background:**

The COVID-19 pandemic has raised awareness of infection prevention and control. We found that the incidence of nosocomial infection in neurosurgery has changed. This study aimed to evaluate the impact of “coronavirus disease 2019 (COVID-19) prevention and control measures” on nosocomial infections in neurosurgery.

**Methods:**

To explore changes in nosocomial infections in neurosurgery during the COVID-19 pandemic, the clinical data of inpatients undergoing neurosurgery at Taizhou Hospital of Zhejiang Province between January 1 and April 30, 2020 (COVID-19 era) were first analyzed and then compared with those from same period in 2019 (first pre-COVID-19 era). We also analyzed data between May 1 and December 31, 2020 (post-COVID-19 era) at the same time in 2019 (second pre-COVID-19 era).

**Results:**

The nosocomial infection rate was 7.85% (54/688) in the first pre-COVID-19 era and 4.30% (26/605) in the COVID-19 era (*P* = 0.01). The respiratory system infection rate between the first pre-COVID-19 and COVID-19 eras was 6.1% vs. 2.0% (*P* < 0.01), while the urinary system infection rate was 1.7% vs. 2.0% (*P* = 0.84). Between the first pre-COVID-19 and COVID-19 eras, respiratory system and urinary infections accounted for 77.78% (42/54) vs. 46.15% (12/26) and 22.22% (12/54) vs. 46.15% (12/26) of the total nosocomial infections, respectively (*P* < 0.01). Between the second pre-COVID-19 and post-COVID-19 eras, respiratory system and urinary accounted for 53.66% (44/82) vs. 40.63% (39/96) and 24.39% (20/82) vs. 40.63% (39/96) of the total nosocomial infections, respectively (*P* = 0.02).

**Conclusions:**

The incidence of nosocomial infections in neurosurgery reduced during the COVID-19 pandemic. The reduction was primarily observed in respiratory infections, while the proportion of urinary infections increased significantly.

## Background

Since the end of 2019, patients with coronavirus disease has emerged as a new infection [1]. Along with the spread of the outbreak, the coronavirus disease 2019 (COVID-19) pandemic has become the worst global health emergency in history.

Severe acute respiratory syndrome coronavirus 2 (SARS-CoV-2) (the disease was named COVID-19) replicates mainly in the upper and lower respiratory tract [2] and its transmission occurs primarily through respiratory droplets and aerosols [3]. SARS-CoV-2 can be transmitted by asymptomatic infected individuals [4], it is contagious during the latency period [5], and this causes the high levels of infectivity of COVID-19.

Based on the knowledge of the characteristics and transmission routes of the COVID-19 infection, hospitals in China have developed the corresponding prevention and effective strategies to control the nosocomial transmission of COVID-19.

Nosocomial infections are main complications of neurosurgery. The most common infectious complications of the neurological intensive care unit are pneumonia, urinary tract infections, bloodstream infections, and intracranial infections (meningitis and ventriculitis) [6]. Nosocomial pneumonia is associated with a prolonged stay and increased in-hospital death [7]. Nosocomial infections also increase hospital costs through the additional use of drugs and by increasing the length of hospital stay [8].

The COVID-19 pandemic has raised awareness of infection prevention and control strategies [9]. Hospitals at all levels have strengthened infection control, and we found that the incidence of nosocomial infection in neurosurgery has changed. However, we do not know exactly how this happened. To our knowledge, previous studies have not reported data on the prevalence of nosocomial infections in neurosurgery as a consequence of the COVID-19 pandemic. Therefore, we analyzed the clinical data of inpatients undergoing neurosurgery of a Chinese Tertiary-Care Hospital between 2019 and 2020, aimed to evaluate the impact of “COVID-19 prevention and control measures” on nosocomial infections in neurosurgery.

## Methods

### Study design

To explore the changes in nosocomial infections in neurosurgery during the COVID-19 pandemic, we analyzed the clinical data of inpatients undergoing neurosurgery at Taizhou Hospital of Zhejiang Province between January 1 and April 30, 2020, the peak COVID-19 era. The data were compared with those from the same period in 2019. We also compared the data between May 1 and December 31, 2020, with the data from the same period in 2019.

We established January 1, 2020, as the turning point because patients with pneumonia related to the South China Seafood Market (suspected COVID-19) were transferred to Wuhan Jinyintan Hospital (Wuhan Infectious Disease Hospital) for isolation and treatment on December 31, 2019. Visitors or family members were not allowed to visit the hospitals. We established the end of April 2020 as another turning point because the Wuhan lockdown was lifted on April 8, 2020, marking a stage victory in China’s COVID-19 prevention and control efforts. After May 2020, the entire country gradually resumed normal production and life.

The diagnostic criteria for “nosocomial infection” included infections that arise in any inpatient or outpatient setting and appear 48 h after hospitalization, or within 30 days after receiving health care, or up to 90 days after undergoing certain surgical procedures, these criteria were based on the diagnostic criteria for hospital infection established by the Ministry of Health of the People’s Republic of China [10]. In order to effectively ensure the accuracy, nosocomial infection are reported by clinicians and confirmed by staff members of the hospital’s infection control department.

### Data collection

There were 258 counts including 16 with multiple infections among 242 patients of nosocomial infection in the neurosurgery department (including 9 neurosurgical ICU beds and 46 general beds) of the Taizhou Hospital of Zhejiang Province, between January 2019 and December 2020. These cases were included in this study as research participants and retrospectively analyzed. All data were obtained from the Taizhou Hospital nosocomial infection system, which is a web-based data system, and the system data was checked by both the doctors and the staff of the hospital infection control department. Among 242 study patients, 114 were men, and 128 were women, with an average age of 60.69 ± 14.39 years. We analyzed the clinical data of the patients, including sex, age, length of hospital stay (including time spent in neurosurgery and later in rehabilitation), emergency craniotomy, period of nosocomial infection (before or after the COVID-19 pandemic), site of infection, medical records, laboratory reports, and surgical records. The distribution of infection sites and changes in nosocomial infection rates in neurosurgical departments before and after the COVID-19 pandemic were statistically analyzed, possible explanations were also explored. Additionally, we analyzed changes in the use of liquid soap and hand disinfectants in neurosurgery before and after the COVID-19 pandemic. All experimental protocols were approved by the Ethics Committee of the Taizhou Hospital of Zhejiang Province (No. K20211123).

### Statistical analysis

The incidence of nosocomial infection, the site of nosocomial infection and the use of liquid soap or hand disinfectant in neurosurgery before and after the COVID-19 pandemic were analyzed using SPSS 22.0. Normal distribution measurement data were expressed as mean ± standard deviation (x ± s). The four periods were then compared using the t-tests and χ^2^ tests. Statistical significance was set at P < 0.05.

## Results

During the study period, 258 counts with nosocomial infections were enrolled in the study. They were divided into four periods: period 1 first pre-COVID-19 era (1/1/2019–4/30/2019); period 2 s pre-COVID-19 era (5/1/2019–12/31/2019); period 3 COVID-19 era (1/1/2020–4/30/2020); and period 4 post-COVID-19 era (5/1/2020–12/31/2020). The mean age of the patients in the COVID-19 era was higher than that in the first pre-COVID-19 era. The mean length of hospital stay in the COVID-19 era (31.29 ± 26.09 days) was shorter than in the first pre-COVID-19 era (36.20 ± 31.90 days). No significant differences in age, sex, length of hospital stay, and operation were observed between the COVID-19 era and first pre-COVID-19 era (*P* > 0.05) (Table 1).

**Table 1:** Characteristics of nosocomial infection patients during the year of 2020 compared with the year of 2019

Periods	Period 1	Period 2	Period 3	Period 4	*P*1	*P*2	*P*3
Characteristics	First Pre-COVID-19 era January 2019–April 2019 (n = 54) Mean ± SD or n (%)	Second Pre-COVID-19 era May 2019–December 2019 (n = 82) Mean ± SD or n (%)	COVID-19 era January 2020–April 2020 (n = 26) Mean ± SD or n (%)	Post-COVID-19 era May 2020–December 2020 (n = 96) Mean ± SD or n (%)	Period 1 vs. Period 3	Period 2 vs. Period 4	Period 2 vs. Period 3
Age, year	56.94 ± 17.82	60.99 ± 11.78	59.65 ± 15.70	63.23 ± 13.03	0.51	0.23	0.65
Sex, n (%)					0.48	0.97	1.00
Male	30 (55.6)	37 (45.1)	12 (46.2)	43 (44.8)			
Female	24 (44.4)	45 (54.9)	14 (53.8)	53 (55.2)			
Length of stay, d	36.20 ± 31.90	39.10 ± 38.07	31.29 ± 26.09	25.28 ± 19.55	0.50	< 0.01	0.33
Operation, n (%)					0.06	0.06	0.52
Emergency operation	34 (63.0)	57 (69.5)	15 (57.7)	50 (52.1)			
Elective operation	1 (1.9)	8 (9.8)	4 (15.4)	17 (17.7)			
No operation	19 (35.2)	17 (20.7)	7 (26.9)	29 (30.2)			

However, the mean length of hospital stay in the post-COVID-19 era was significantly shorter than in the second pre-COVID-19 era (25.28 ± 19.55 days vs. 39.10 ± 38.07 days; *P* = 0.002). No significant differences in age, sex, and operation were observed between the post-COVID-19 and second pre-COVID-19 eras (Table 1).

**Fig. 1:**
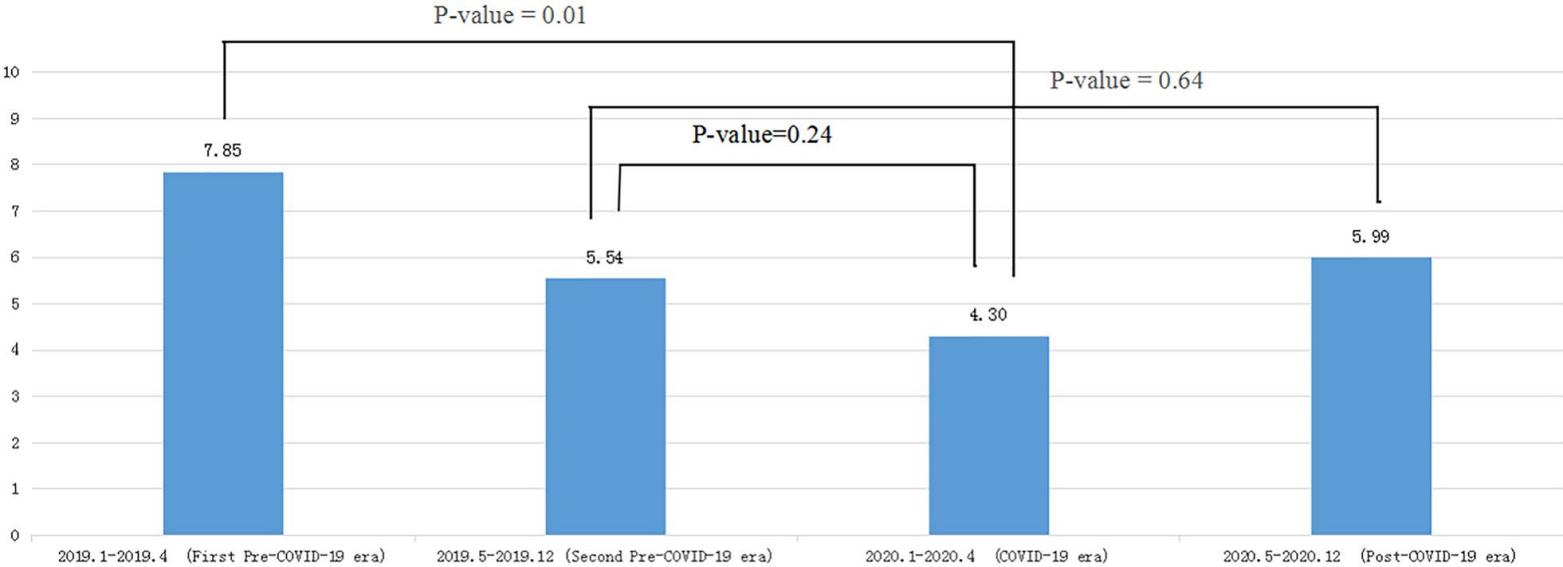
The rate of nosocomial infection in different periods

The nosocomial infection rate in the neurosurgery department was 7.85% (54/688) in the first pre-COVID-19 era and 4.30% (26/605) in the COVID-19 era in the neurosurgery department. The prevalence of nosocomial infections differed significantly between the two periods (*P* = 0.01) (Fig. 1). The prevalence of nosocomial infections was 5.54% (82/1479) in the second pre-COVID-19 era compared with 5.99% (96/1603) in the post-COVID-19 period. There was no statistical difference in the prevalence of nosocomial infections between the second pre-COVID-19 era and post-COVID-19 (*P* = 0.64), or between the second pre-COVID-19 and COVID-19 era (*P* = 0.24) (Fig. 1).

The nosocomial infection sites primarily comprised respiratory system, urinary system, central nervous system infection, and others (e.g., skin, blood system, gastrointestinal system, and otitis media). In the first pre-COVID-19 era, the respiratory system and urinary system infection rates were 6.1% (42/688) and 1.7% (12/688), respectively. In the COVID-19 era however, the respiratory system and urinary system infection rates were 2.0% (12/605) and 2.0% (12/605), respectively. Due to the impact of the COVID-19 intervention, we observed that the nosocomial infection rate of the respiratory system had significantly decreased (*P* < 0.01) between the COVID-19 and first pre-COVID-19 eras, but the rate of urinary infections did not change significantly (*P* = 0.84) (Table 2).

**Table 2:** Infection rate of nosocomial infection sites in different periods

Periods	Period 1	Period 2	Period 3	Period 4	*P*1	*P*2	*P*3
Characteristics	First Pre-COVID-19 era January 2019–April 2019 (688) n (%)	Second Pre-COVID-19 era May 2019–December 2019 (1479) n (%)	COVID-19 era January 2020–April 2020 (605) n (%)	Post-COVID-19 era May 2020–December 2020 (1603) n (%)	Period 1 vs. Period 3	Period 2 vs. Period 4	Period 2 vs. Period 3
Respiratory system	42 (6.1)	44 (3.0)	12 (2.0)	39 (2.4)	< 0.01	0.37	0.23
Urinary system	12 (1.7)	20 (1.4)	12 (2.0)	39 (2.4)	0.84	0.04	0.33
Neurosurgical system	0 (0)	13 (0.9)	0 (0)	6 (0.4)	/	0.11	/
Others	0 (0)	5 (0.3)	2 (0.3)	12(0.7)	0.22	0.15	1.00

However, infection rate of the respiratory system between the second pre-COVID-19 era and post-COVID-19, was 3.0% (44/1479) vs. 2.4% (39/1603); thus, there was no significant differences (*P* = 0.37). Meanwhile, the urinary system infection rate was 1.4% (20/1479) vs. 2.4% (39/1603), with a statistically significant difference (*P* = 0.04) (Table 2). Between the second pre-COVID-19 and COVID-19 eras, the respiratory system infection rate was 3.0% (44/1479) vs. 2.0% (12/605), showing no significant differences (*P* = 0.23). Meanwhile, the infection rate of the neurosurgical system was 0.9% (13/1479) vs. 0% (0/605) (Table 2).

**Fig. 2:**
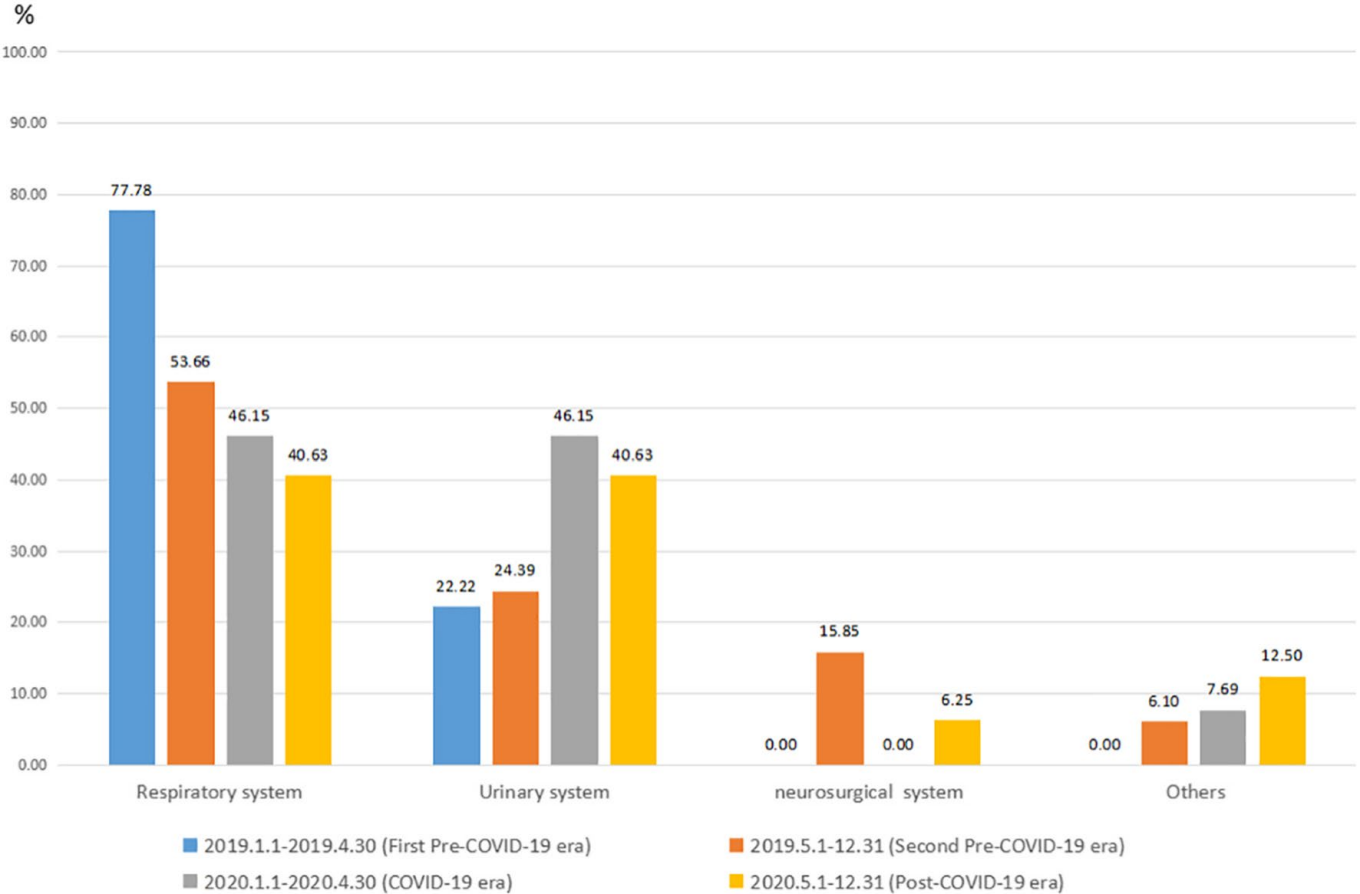
The proportion of nosocomial infection sites in different periods (%)

In the first pre-COVID-19 era, compared to their occurrence in the COVID-19 era, respiratory system nosocomial infections accounted for 77.78% (42/54) vs. 46.15% (12/26) of the total the nosocomial infections, while urinary system infections accounted for 22.22% (12/54) vs. 46.15% (12/26) of the in total nosocomial infections; these differences were statistically significant (*P* = < *0.01*). Furthermore, a comparison of the second pre-COVID-19 era with the post-COVID-19 era also revealed the same trend: respiratory system infections accounted for 53.7% (44/82) vs. 40.6% (39/96) of the total nosocomial infections; and urinary infections accounted for 24.4% (20/82) vs. 40.6% (39/96) of the total nosocomial infections; these differences were statistically significant (*P* = 0.02). Due to the impact of COVID-19 intervention strategies, the proportion of respiratory infections in nosocomial infections has decreased significantly, while the proportion of urinary infections has increased. The incidence of nosocomial respiratory infections declined continuously over periods 1 to 4. (Fig. 2).

**Fig. 3:**
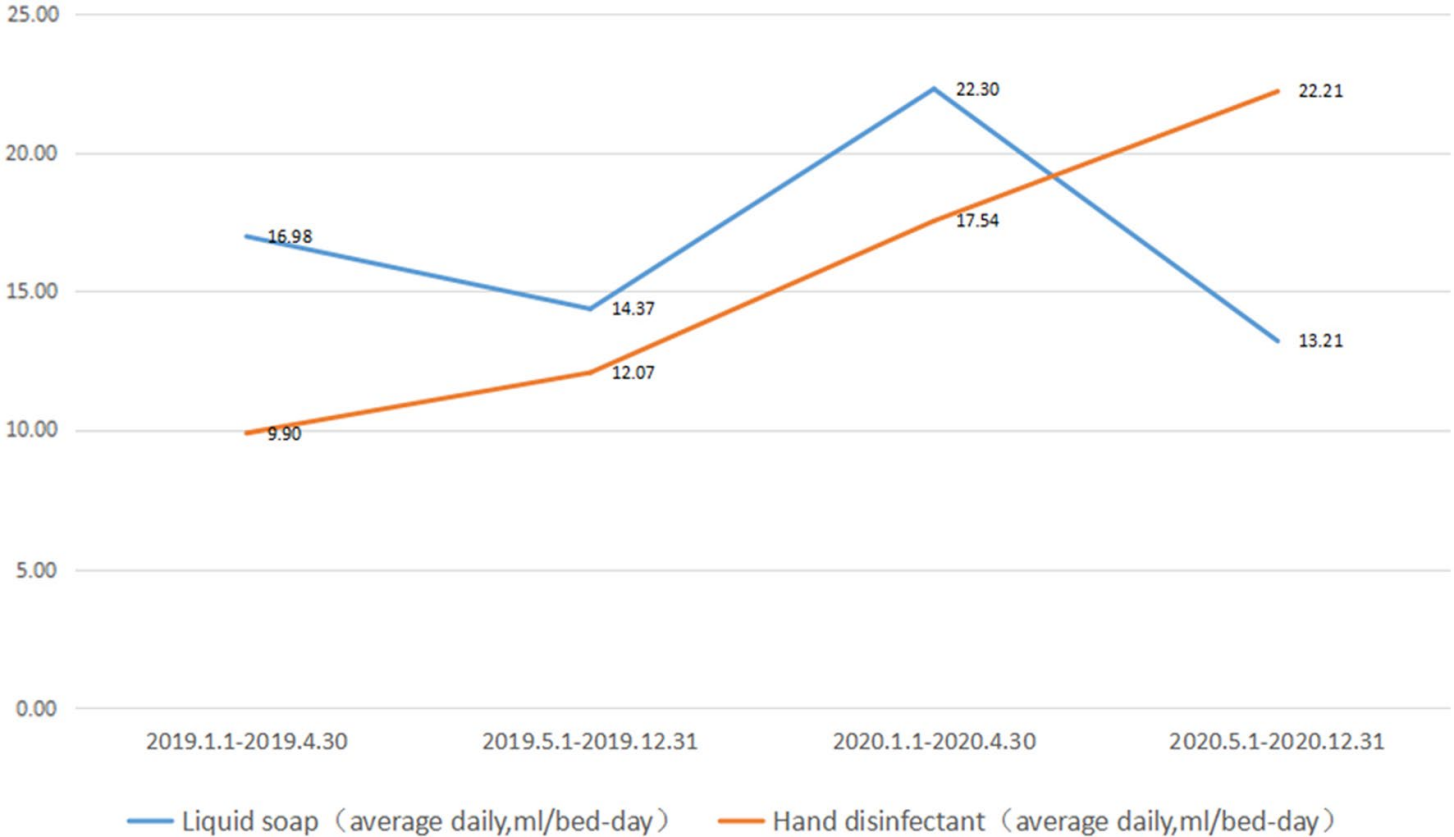
Use of liquid soap and hand disinfectant in different periods

Hand hygiene is considered the most effective way to control nosocomial infections. The use of liquid soap and hand disinfectants reflects hand hygiene, therefore, we analyzed the use of these two items before and after COVID-19. In the first pre-COVID-19 era, the use of liquid soap and hand disinfectant was 16.98 ml/bed-day and 9.9 ml/bed-day, respectively (total 26.88 ml/bed-day). While, in the COVID-19 era, the use of liquid soap and hand disinfectant was 22.30 ml/bed-day and 17.54 ml/bed-day, respectively (total 39.84 ml/bed-day). On comparing the second pre-COVID-19 era with the post-COVID-19, the use of liquid soap and hand disinfectant, respectively, was 14.37 ml/bed-day and 12.07 ml/bed-day (total 26.44 ml/bed-day) vs. 13.21 ml/bed-day and 22.21 ml/bed-day (total 35.42 ml/bed-day) (Fig. 3).

## Discussion

COVID-19 has emerged as a new infection and began to infiltrate modern society at the beginning of 2020 [1, 11]. It has changed people’s lifestyle and medical behavior. A significant decrease in the number of pediatric admissions in 2020 was observed in many countries, including Finland, the United States, and Morocco [12–15]. Italian doctors reported a reduced rate of surgical site infections during the COVID-19 pandemic compared with previous years [16]. This study revealed that simple and easily viable precautions, such as wearing surgical masks and restricting visitors, have emerged as promising tools for reducing the risk of surgical site infections [16]. A significant decrease in hospital-acquired infections and antibiotic prescriptions during the COVID-19 pandemic compared to that in the preceding year may be largely attributable to the huge increase in hygiene measures [17].

Little research has been conducted on the relationship between COVID-19 intervention and nosocomial infections and nosocomial neurosurgical infections. In this study, we first observed that nosocomial infections in the neurosurgery department dropped by 45% [(7.85–4.30%)/7.85%] after the response to the COVID-19 pandemic compared with their frequency in the same period in 2019. We also divided 2019 and 2020 into four periods based on the outbreak and development of COVID-19 in China and conducted a comparative study. We consider the COVID-19 era as the primary research object and the post-COVID-19 era as the secondary research object. The prevalence of nosocomial infections differed significantly among the four periods (*P* < 0.05).

When we further studied the location of nosocomial infections, we found the most significant (COVID-19 era vs. first pre-COVID-19 era; P < 0.01; Table 2) decrease in respiratory infections, which was related to health habits during the COVID-19 period. Masks are widely discussed during the ongoing COVID-19 pandemic, but masks alone may not have been greatly attributable to the decreased spread of the COVID-19 pandemic unless coupled with adequate social distancing, diligent hand hygiene, and other proven prevention measures [18]. During the COVID-19 epidemic, masks, gloves, and hand sanitizers were used to prevent transmission because respiratory droplets and contact transmission are the main routes of transmission of this disease [19]. Our analysis showed that the amount of hand sanitizer used was directly proportional to the decrease in respiratory infections.

Additionally, we found that the total proportion of total nosocomial infections in the urinary system increased during and after the COVID-19 pandemic, this was accompanied by a decrease in the proportion of respiratory system nosocomial infections (Fig. 2). This indicates that the prevention and control measures of COVID-19 cannot reduce the incidence of urinary system infections (Table 2). Many surgical procedures are time-sensitive, but they are not necessarily nonelective, patients treated sooner may benefit the most from surgical intervention [20]; Age and preoperative length of hospital stay are risk factors for postoperative central nervous system infection [21, 22]. We also found that the infection rate of the neurosurgical system between the second pre-COVID-19 era and COVID-19 era, was 0.9% vs. 0%, this may indicate that the prevention and control measures for COVID-19 can not reduce the incidence of neurosurgical system infection. In this study, we did not find significant differences in other infections between the four periods (Table 2).

Our study identified a significant increase in the daily use of liquid soap and hand disinfectants per day in the COVID-19 era compared to their usage in the first pre-COVID-19 era. This reflects the efforts made for COVID-19 prevention and control. This also shows that the COVID-19 pandemic has improved hand hygiene among the medical staff. In the post-COVID-19 period, the use of liquid soap reduced compared with that during COVID-19 era, but the use of hand disinfectants continued to increase as the total amount increased, suggesting that medical staff used hand disinfectants more frequently than they used liquid soap in the post-COVID-19 period.

These results indicate that intervention during the COVID-19 era can significantly reduce the nosocomial infection rate (*P* < 0.01), especially respiratory infections (*P* < 0.01). Therefore, we believe that relevant measures should be maintained in future studies. We also found that urinary system infection increased after COVID-19; we believed this is due to that medical staff paid too much attention to respiratory-related defenses (such as masks). Further steps are needed to reduce the incidence of urinary system infections.

## Limitation

This study has several limitations. First, the data were obtained from a single tertiary hospital in China, so the applicability of these findings may not apply to all hospitals. Second, we did not analyze the etiology of nosocomial infections in different periods, so we did not know the changes in the pathogens. Third, we did not conduct a further study on catheter-related nosocomial infections, which might have led to a bias toward respiratory tract infections and urinary tract infections. Finally, we did not consider whether the use of bar soap could have influenced the amount of liquid soap used, which might have biased our results.

## Conclusions

COVID-19 intervention reduced the incidence of nosocomial infections in neurosurgery, the main reduction being respiratory infections, while the proportion of urinary infections in the total nosocomial infections increased significantly.

## Data Availability

All data generated or analyzed during this study are included in this published article and its additional information files.

## Abbreviations

COVID-19: Coronavirus disease 2019
SARS-CoV-2: Severe acute respiratory syndrome coronavirus 2
